# Simulator sickness when performing gaze shifts within a wide field of view optic flow environment: preliminary evidence for using virtual reality in vestibular rehabilitation

**DOI:** 10.1186/1743-0003-1-14

**Published:** 2004-12-23

**Authors:** Patrick J Sparto, Susan L Whitney, Larry F Hodges, Joseph M Furman, Mark S Redfern

**Affiliations:** 1 Department of Physical Therapy, University of Pittsburgh, Pittsburgh, PA, USA; 2 Department of Otolaryngology, University of Pittsburgh, Pittsburgh, PA, USA; 3 Department of BioEngineering, University of Pittsburgh, Pittsburgh, PA, USA; 4 Department of Computer Science, University of North Carolina-Charlotte, Charlotte, NC, USA

**Keywords:** VR, balance, physical therapy, virtual environment, CAVE

## Abstract

**Background:**

Wide field of view virtual environments offer some unique features that may be beneficial for use in vestibular rehabilitation. For one, optic flow information extracted from the periphery may be critical for recalibrating the sensory processes used by people with vestibular disorders. However, wide FOV devices also have been found to result in greater simulator sickness. Before a wide FOV device can be used in a clinical setting, its safety must be demonstrated.

**Methods:**

Symptoms of simulator sickness were recorded by 9 healthy adult subjects after they performed gaze shifting tasks to locate targets superimposed on an optic flow background. Subjects performed 8 trials of gaze shifting on each of the six separate visits.

**Results:**

The incidence of symptoms of simulator sickness while subjects performed gaze shifts in an optic flow environment was lower than the average reported incidence for flight simulators. The incidence was greater during the first visit compared with subsequent visits. Furthermore, the incidence showed an increasing trend over the 8 trials.

**Conclusion:**

The performance of head unrestrained gaze shifts in a wide FOV optic flow environment is tolerated well by healthy subjects. This finding provides rationale for testing these environments in people with vestibular disorders, and supports the concept of using wide FOV virtual reality for vestibular rehabilitation.

## Background

One out of three elderly persons and more than one out of five working adults report dizziness[1,2]. There is a growing body of literature that suggests that persons with dizziness due to vestibular disorders fall, regardless of age [3]. Falls in persons with vestibular disorders have potentially catastrophic consequences[4]. Thus, development of rehabilitation methodologies that can improve balance could have a great impact on public health.

The use of virtual reality (VR) has been explored in many areas of physical and mental rehabilitation [5-8]. Viirre [9,10] and Kramer et al. [11] were the first to discuss the use of VR with persons with vestibular disorders. The theoretical basis for using VR in the treatment of vestibular disorders is two-fold. First, persons with peripheral vestibular disorders have disequilibrium and complain of visual blurring [12]. These common symptoms may be caused by abnormalities in the vestibulo-ocular reflex (VOR) gain during head movements. Functional recovery of the VOR requires both visual inputs and movements of the head and body [13]. Retinal slip, i.e. movement of a visual image across the retina, is a powerful signal that can induce adaptation of vestibular responses [14]. If care is taken to minimize delays between head tracking devices and image updates, VR-induced retinal slip can be delivered in a controlled manner in order to cause adaptation. A randomized trial has demonstrated that persons with uncompensated peripheral vestibular disorders can improve with vestibular rehabilitation directed at inducing retinal slip [15]. People can also adapt to vestibular injuries through movement. Shepard et al. were able to reduce symptoms in 87% of patients who had chronic unilateral peripheral vestibular loss for at least 2 months [16]. Therefore, exposure to visual experiences and movement are key to the functional recovery of persons with vestibular disorders.

Secondly, people with vestibular disorders complain of what has been called "space and motion discomfort" (SMD) and "visual vertigo" [17,18]. Situations that have been reported to precipitate SMD or visual vertigo include: walking in supermarket aisles or shopping malls, movement in cars and trains, long visual distances, or complex and confusing visual stimuli. Consequently, VR could allow persons with vestibular disorders to experience graded exposure to symptom-provoking situations in a controlled environment. Using VR for exposure therapy has a well-established foundation in the treatment of specific phobias (e.g. fear of heights) [8,19].

Wide field of view (FOV), screen-based projection devices (or spatially immersive displays) originated with the CAVE™ at the University of Illinois in Chicago [20]. Although the space required and cost make these systems impractical for clinical use, their wide FOV allow research laboratories to investigate how different motion cues affect balance and vestibular rehabilitation. One advantage of wide FOV devices is their ability to provide motion cues in the periphery, which can result in a greater sense of vection, or self-movement, compared with more limited FOV devices [21]. We believe that these factors may provide a substantial benefit compared with narrower FOV devices such as HMDs in the treatment of vestibular disorders. However, the wide FOV devices have also been associated with greater reports of simulator sickness [22]. Thus, while a wide FOV is desirable from a theoretical standpoint because a greater perception of motion occurs in the periphery, this same factor may elevate levels of simulator sickness and may be cause for discontinuing a treatment.

The primary purpose of this paper is to present preliminary evidence for the ability of subjects to tolerate gaze shifting while situated in a wide FOV optic flow environment. We will demonstrate that healthy subjects were able to tolerate the environments without having a large incidence in simulator sickness. The incidence of simulator sickness depended strongly on how much experience the subjects had in the environment, and weakly on the duration of exposure within each visit.

## Methods

### Subjects

Nine adults (22–75 years, mean ± S.D. 39 ± 19 yrs) with no history of vestibular system pathology participated after providing informed consent. Subjects had a visual acuity of 0.3 LogMAR units or better without using corrective lenses, and contrast sensitivity greater than 1.8 (Pelli-Robson Contrast Sensitivity). The protocol was approved by the University of Pittsburgh Institutional Review Board.

### Equipment

**Figure 1:**
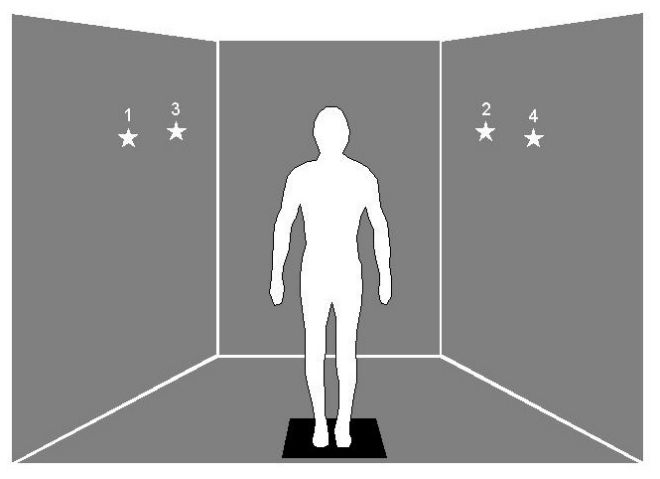
Experimental set-up for Task H, Visit 1 (see Tables 1 and 2 for explanation). Subjects stood upright on force platform and performed gaze shifts while target moved on a solid background. The target moved every 3 to 6 seconds from positions 1 to 4, located 40 to 50 degrees from midline.

The Balance NAVE Automatic Virtual Environment (BNAVE), a wide field of view projection-based immersive display system, was developed to investigate the multi-sensory interactions in postural control [23]. Three 2.4 m × 1.8 m (vertical × horizontal) back-projected screens are arranged as shown in Figure 1. The side screens make an included angle of 110° with the front screen. The front screen is 1.5 m from the user, and the opening of the BNAVE at the location of the subject is approximately 2.9 m. The images are displayed using Epson 810p PowerLite LCD monoscopic projectors, with a pixel resolution of 1024 × 768 for each screen. Each projector is connected to an NVIDIA GeForce4 graphics processing unit (64 MB texture memory) installed in a separate PC (Pentium, 2.2 GHz, 512 MB RAM) running Windows 2000. The movement of the images on the three PCs is synchronized and controlled by a server via a local area network. The update rate of the images is consistently at least 30 frames per second.

**Figure 2:**
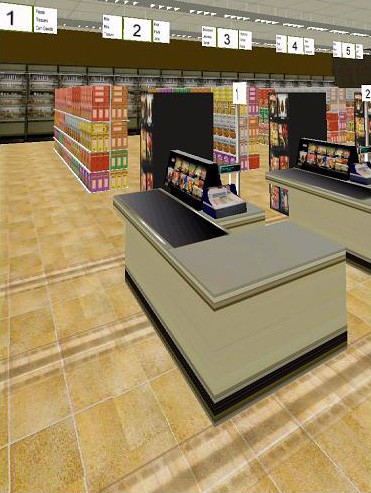
Virtual Grocery Store developed for providing exposure therapy for patients with dizziness that is increased in similar environments. Aisle length, shelf height, the number of products on the shelves, and object textures can all be manipulated depending on the goal of the therapy session.

Several environments can be used for vestibular rehabilitation. One environment produces optic flow through the use of moving geometric patterns such as stripes or squares of alternating colors. Scene characteristics such as the spatial frequency, contrast, direction and speed of movement can all be independently prescribed. Furthermore, targets can be inserted into the environment that subjects are requested to look at. Consequently, for vestibular rehabilitation, we can ask a patient to perform unrestrained head gaze shifts to acquire moving targets while a background of moving stripes is moving past the patient, simulating the functional task of looking for a product while moving down the aisle of a grocery store. A virtual grocery store has also been developed (Figure 2). This environment contains several aisles, each with a different product theme. The dimensions of the aisle (width and length) are adjustable. Scene complexity can be altered by increasing the number of items on the shelves. The objects within the environment have both software-generated and photographic texture maps. In both environments, the task difficulty can be modified by varying the scene characteristics, thus exposing the patient to symptom-producing situations in a controlled and graded manner. In each environment, three-dimensional models were created using 3D Studio Max. Although the projectors used were not stereoscopic, a strong illusion of depth was elicited based on monocular depth cues such as perspective projection. The location of the eyepoint used for the perspective projection was based upon a fixed stance location in the horizontal plane and the subject's eye height. In addition, although head-tracked perspective correction was not used in the current application, this capability is possible and fully functional using a Polhemus Fastrak position tracking system (Polhemus, Inc. Colchester, VT).

### Procedure

The ability of subjects to perform gaze shifts in response to moving targets superimposed on both static and moving backgrounds was examined. Eight different gaze tasks are performed on each visit (Table 1). Each gaze task was performed for 90 s with alternating movements to the left and right every 3 to 6 seconds (except for tasks B, F, H). Each task was performed with the six different backgrounds (Table 2). Each background condition was performed on a different visit. Background conditions 1 and 2 did not have optic flow and thus served as a control conditions for the remaining 4 backgrounds, which were randomized over the next 4 visits. During the high contrast conditions, the luminance of the stripes was 1 and 170 cd/m^2^, respectively. During the low contrast conditions, the luminance of the stripes was 15 and 34 cd/m^2^. The low contrast condition was based on average measurements of luminance obtained from products sampled at a local grocery store, using a luminance meter (LS-100 Luminance Light Meter, Minolta Corp. Ramsey, NJ). The spatial frequencies were set according to common sizes of soup cans (high, 4.2 cycles/meter) and cereal boxes (low, 1.4 cycles/meter) found in the local grocery store. The simulated velocity of the optic flow was 0.5 m/s. The central 25° of the display was masked by a solid region with a luminance of 15 cd/m^2 ^in order to avoid aliasing in the display as the stripes became smaller in the distance.

**Table 1:** Gaze tasks performed on each of the six visits. On trials 3 to 8, the order of tasks D, E, F, G, H, and I are randomized on each visit.

Trial	Task
0	A) Initial reading
1	B) 10 deg head saccades to the right – calibration of head sensor
2	C) Control task – no head or eye movements
3	D) Head center, Eye saccades to ± 10 deg
4	E) Head left 50 deg, Eye saccades to ± 10 deg
5	F) Head right 50 deg, Eye saccades to ± 10 deg
6	G) Gaze stabilization during sinusoidal head movement at 0.25 Hz (VOR)
7	H) Gaze saccades to ± 40 and ± 50 deg from midline
8	I) Smooth pursuit to left between 10 – 60 deg, followed by smooth pursuit to right between 10 – 60 deg

**Table 2:** Background conditions for each of the six visits. On visits 3 to 6, the order of conditions C, D, E, and F are randomized.

Visit	Condition	Optic Flow	Contrast	Spatial Frequency
1	A	No	None	None
2	B	No	High	Low
3	C	Yes	Low	Low
4	D	Yes	Low	High
5	E	Yes	High	Low
6	F	Yes	High	High

**Figure 3:**
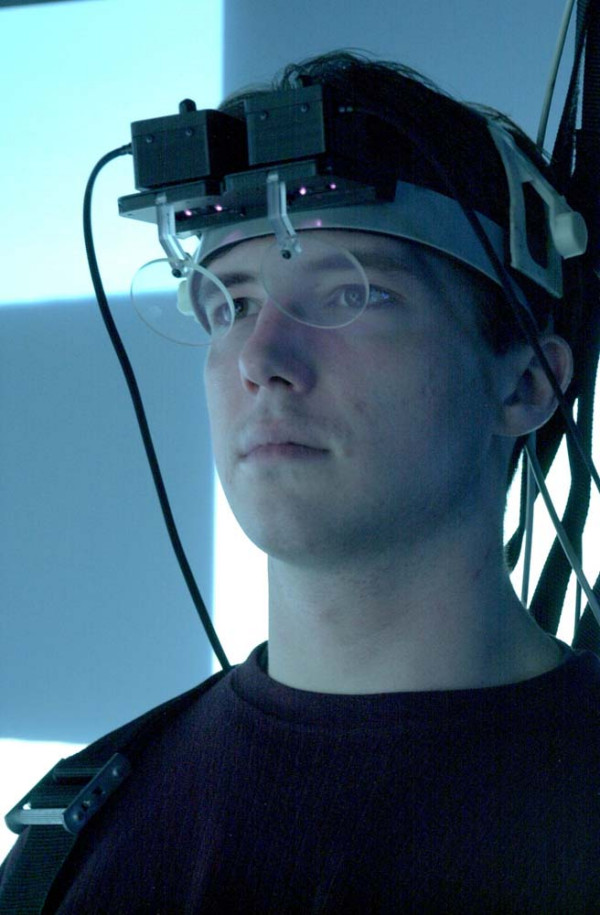
Video-oculography (VOG) see-through goggles used to measure eye-in-head position in the vertical and horizontal planes. Subject also wore 6 degrees of freedom electromagnetic tracker on top of his head to measure head in space position. Both signals are combined to obtain gaze, or eye gaze-in-space position.

Rests of 3 minutes were provided after each task, during which Subjective Units of Discomfort (SUDS, 0–10 range) was rated and the Simulator Sickness Questionnaire (SSQ) was completed [24]. The SSQ contains 16 items on which subjects rate the degree of severity on a 4-item scale (0 = none, 1 = slight, 2 = moderate, 3 = severe). A total score is computed along with 3 subscales – nausea (general discomfort, increased salivation, sweating, nausea, difficulty concentrating, stomach awareness, burping), oculomotor stress (general discomfort, fatigue, headache, eyestrain, difficulty focusing, difficulty concentrating, blurred vision), and disorientation (difficulty focusing, nausea, head fullness, blurred vision, dizzy: eyes open, dizzy: eyes closed, vertigo). Furthermore, pulse and blood pressure was monitored after every trial using an automatic device. Initial recordings of each of the measures were also recorded prior to exposure. During each trial, postural sway was recorded using a force platform and 6 degrees of freedom electromagnetic trackers placed on the head and waist (Polhemus Fastrak, Colchester, VT). The accuracy of the trackers is 0.8 mm in translation and 0.15° in rotation, and the stated resolution of 0.2 mm in translation and 0.025° in orientation. The trackers have a latency of 4 ms and were digitized at 20 Hz. The horizontal and vertical eye movements were measured using video-oculography (VOG, Figure 3). The VOG device (Micromedical, Chatham, IL) was fastened using an adjustable helmet insert and contained see-through dichroic glass that reflected images of the eyes up to infrared cameras. The accuracy of the VOG is 0.3 deg and the images are captured at 60 Hz. Using the sampling rates of the tracker and VOG, the maximum delay between recording simultaneous movements of both the head and eye would be 33 ms. The head and eye movements were calibrated using targets placed in known locations. Eye-in-head position is combined with head-in-space position to yield continuous gaze position (eye-in-space). The timing and accuracy of the head gaze movements with respect to the targets will be the subject of a future report.

### Data Analysis

Five dependent variables of interest were examined: SUDS, total SSQ, and 3 SSQ subscales. The three subscales of the SSQ were computed by summing the scores for the component items of each subscale, and multiplying by an appropriate weighting factor (9.54 for Nausea, 7.58 for Oculomotor, and 13.92 for Disorientation) [24]. The total SSQ score was equal to the sum of the 3 subscales, multiplied by 3.7. Histograms of each dependent variable were plotted according visit number (1 to 6) and trial number (0 to 8). After observing that the data were not normally distributed due to a large majority of 0 responses and the presence of long tails, we tabulated the frequency of non-zero responses for each dependent variable. The effect of visit number and trial number on the frequency of non-zero responses was evaluated using χ^2 ^statistics. Because of the large number of comparisons (5 dependent variables × 2 main effects), the significance level was set at α = 0.05/10 = 0.005.

## Results

**Figure 4:**
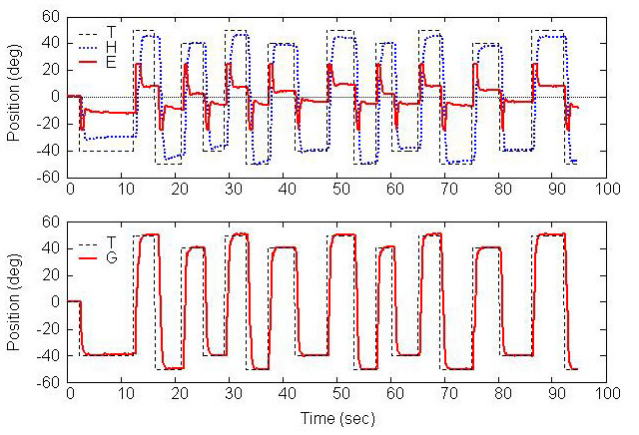
Gaze shifts during Task H obtained from 1 subject. Top: target yaw position (T), head-in-space yaw position (H), and eye-in-head yaw position (E). Bottom: target yaw position (T), and eye gaze-in-space yaw position (G).

Gaze shifts performed by one subject in response to targets moving at least 80° in the yaw plane (Task G) are shown in Figure 4. These gaze shifts are combinations of head and eye movements. The top plot demonstrates the target-in-space yaw position (T), head-in-space yaw position (H), and eye-in-head yaw position (E). The last two quantities are combined to produce the gaze position (G) shown in the bottom plot. In this example, it can be seen that the eye and head movements effectively combine to allow the person to look at the desired target position. Analytical techniques will allow us to quantify head and eye coordination strategies in persons with vestibular disorders, both in optic flow fields and more complex environments.

The tolerance of the subjects to the gaze shifts was assessed using Subjective Units of Discomfort (SUDS) and the Simulator Sickness Questionnaire (SSQ). For each of the measures, the majority of the responses were zero. Therefore, each of the measures was converted into a binary scale consisting of responses equal to zero or greater than zero. The SUDS rating was greater than zero only 25% of the time. For each of the SSQ subscales, a score greater than 0 was given if any of the 7 component items for the subscale was rated greater than 0. The SSQ:Oculomotor subscale had the most non-zero responses, at 29%. The SSQ:Nausea and SSQ:Disorientation subscales had 12% and 5% non-zero responses, respectively. Overall, the SSQ-Total had 31% non-zero responses.

The effect of visit number and trial number on the frequency of non-zero responses was examined using χ^2 ^statistics. The effect of visit number was significant for SUDS, SSQ:Nausea, SSQ:Oculomotor, and SSQ:Total (Table 3). The most obvious finding was that the number of non-zero responses was significantly greater the first visit. The effect of trial number was not significant for all measures (Table 4).

**Table 3:** Incidence of non-zero responses for the self-reported Subjective Units of Discomfort SUDS) and Simulator Sickness Questionnaire (SSQ) subscales and total score, as a function of visit number. Mean incidence, χ^2 ^test of association, and p value are also provided. * indicates significant effect of visit number.

Visit	SUDS	SSQ:Nausea	SSQ:Oculomotor	SSQ:Disorientation	SSQ:Total
1	35	27	56	9	56
2	38	11	33	0	33
3	22	11	26	5	28
4	25	16	19	10	26
5	19	0	26	1	27
6	10	8	14	8	14

Mean	25	12	29	5	31
χ^2^	17.9	25.2	30.7	10.1	25.3
p-value	.003*	<.001*	<.001*	.072	<.001*

**Table 4:** Incidence of non-zero responses for the self-reported Subjective Units of Discomfort (SUDS) and Simulator Sickness Questionnaire (SSQ) subscales and total score, as a function of trial number. Mean incidence, χ^2 ^test of association, and p value are also provided. No significant effect of trial number was found.

Trial	SUDS	SSQ:Nausea	SSQ:Oculomotor	SSQ:Disorientation	SSQ:Total
0 (initial)	4	7	11	0	11
1	17	6	15	2	15
2	24	9	24	11	31
3	28	13	28	2	30
4	30	13	31	6	33
5	26	15	35	6	37
6	30	15	39	7	41
7	32	15	38	8	40
8	34	17	40	8	40

Mean	25	12	29	5	31
χ^2^	15.0	5.2	16.0	8.0	16.2
p-value	.06	.73	.04	.43	.04

## Discussion

The ability to perform coordinated gaze movements within an optic flow environment may lead to the development of tools to improve outcomes in vestibular rehabilitation. The current research represents the first attempt to assess self-reported tolerance to these movements in a wide field of view environment. The ratings indicate that on a majority of the trials, this group of healthy subjects experienced no discomfort and simulator sickness while performing 8 different types of gaze movements under different optic flow conditions. On 75% of the trials, subjects reported no subjective discomfort. On 69% of the trials subjects reported no symptoms of simulator sickness.

Although there is no data with which to compare the incidence of symptoms during performance of coordinated eye and head movements in an optic flow environment, we have chosen to review the data obtained from flight simulators and head mounted display units. The incidence of symptoms in the current study is at the lower end of the range relative to the previous flight simulator based studies. For example, the incidence of simulator sickness in this military pilots has ranged from 6–62%, depending on the type of simulator [25]. The incidence of simulator sickness after VR exposure in non-pilots is slightly greater; approximately 60 to 80% of subjects report symptoms of eyestrain, headache, nausea, and malaise after only 10–20 minutes [26,27]. The decreased incidence of problems may be related to several factors. For one, the within trial exposure time was short, approximately 90 seconds. Therefore the total exposure time was only about 12 minutes, which is on the lower end of reported exposures described in the literature [26,28]. Secondly, significant rest breaks were provided between trials. The incorporation of rest breaks to reduce simulator sickness has not been studied well. Thus, the type of display device, as well as the content and nature of the task may have an effect on the amount of sickness. Thus, although the current results are not directly comparable to the previous research, they will serve as a foundation for future work that examines the incidence of symptoms while performing coordinated eye and head movement tasks within a virtual grocery store, or using a head mounted display.

There was a significant effect of visit number of the number of non-zero responses. Analysis of the data revealed that subjects appeared to have greater levels of discomfort and symptoms of simulator sickness on the first visit. It is possible that subjects had greater levels of discomfort due to their lack of prior exposure to the environment/experiment. Furthermore, our data is consistent with findings from other studies that subsequent exposures to environments result in lower simulator sickness [26,28]. Interpretation of this finding is clouded by the confounding effect of the background displayed during visit 1. During the first visit, the subjects always experienced movement of targets superimposed on a solid background without optic flow. The experiment was designed in this way because we assumed that this background would elicit the least amount of symptoms, and would serve as a suitable background for subjects to learn the movements. Consequently, the finding of decreased tolerance to the movements during the first visit was unexpected. Unfortunately, we are not able to distinguish if the increased discomfort and simulator sickness was due to the subject's inexperience with the environment or due to the type of background.

We did not find a significant effect of trial number on the number of non-zero responses to SUDS and the SSQ. However, it was apparent that there was a trend for greater number of non-zero responses as trial number increased for the SUDS, SSQ:Oculomotor, and SSQ:Total Severity. In previous reports using flight simulators, the level of simulator sickness increased as the duration of exposure increased [28]. Moreover, symptoms tended to persist after the simulation was finished [25,29]. Addition of more subjects may reveal the trial effect to be significant. Nonetheless, the short duration of exposure within each trial (i.e. 90 seconds) and the amount of rest provided to the subjects between trials (i.e. 3 minutes) may have ameliorated the development of symptoms as the trials accumulated.

The SSQ:Oculomotor subscale had the greatest number of non-zero responses. Usually, this subscale is elevated secondary to the effects of using a head-mounted display (HMD) device. HMD users frequently suffer from eyestrain, and blurred vision and short-term changes in binocular vision possibly due to alterations in the balance between the vergence and accommodation systems [30,31]. In our case, we attribute the scores to the video-oculography (VOG) device. Several subjects commented that the VOG caused eyestrain. Furthermore, some subjects reported that the dichroic lenses interfered with their viewing of the environment. We intend to examine if using electro-oculography (EOG), which measures eye movements via surface electrodes surrounding the eyes, will reduce the amount of oculomotor symptoms.

The ability to move one's head and search for targets is a functional task that is often impaired in people with vestibular disorders. In vestibular rehabilitation, patients are encouraged to move their head during daily activities because movement is needed for adaptation and reweighting of the sensory signals. Using optic flow and/or virtual environments, this activity could be restored. Our preliminary analysis demonstrates that healthy people are able to coordinate their head and eye movements in the presence of stationary and moving backgrounds with few side effects. The next step is to perform similar experiments with people who have vestibular disorders.

In addition, it is imperative to study if these movements can be tolerated while using narrower FOV HMDs. Head mounted displays will most likely be the desired display system of choice for vestibular rehabilitation because of the relatively modest cost and high portability. However, there are other characteristics of the display systems that need to be considered. For example, it is common for users of HMDs to complain of eyestrain, blurred vision, headache, and nausea [30,31]. In addition, DiZio and Lackner suggest that wearing an HMD effectively increases the mass and inertia of the head, thereby leading to a sensory rearrangement that may have some part in simulator sickness [32]. This theory is supported by the work of Howarth and Finch, who examined the amount of nausea generated while subjects wore an HMD under 2 conditions [33]. In one, subjects changed heading by using a handheld input device. In the other, subjects changed heading by rotating their head. Nausea was significantly greater when subjects navigated using their head. The lag between head movement and scene movement, and the variability in frame update rate has also been considered to play an important role in generating sickness with the use of HMDs [26,33]. However, as head tracking technology has improved, and update lags have been reduced, this factor is probably not as important as it once was. Thus, research on the use of HMDs in people with vestibular disorders is necessary to determine if they can be safely used in this population.

## Conclusion

The performance of head unrestrained gaze shifts in a wide FOV optic flow environment is tolerated well by healthy subjects. This finding provides rationale for testing these environments in people with vestibular disorders, and supports the concept of using wide FOV virtual reality for vestibular rehabilitation.

## References

[B1] Grimby A, Rosenhall U (1995). Health-related quality of life and dizziness in old age.[see comment]. Gerontology.

[B2] Yardley L, Owen N, Nazareth I, Luxon L (1998). Prevalence and presentation of dizziness in a general practice community sample of working age people.[see comment]. Br J Gen Pract.

[B3] Whitney SL, Hudak MT, Marchetti GF (2000). The dynamic gait index relates to self-reported fall history in individuals with vestibular dysfunction. J Vestib Res.

[B4] Tinetti ME, Williams CS (1998). The effect of falls and fall injuries on functioning in community-dwelling older persons. Journals of Gerontology Series A, Biological Sciences & Medical Sciences.

[B5] Sveistrup H, McComas J, Thornton M, Marshall S, Finestone H, McCormick A, Babulic K, Mayhew A (2003). Experimental studies of virtual reality-delivered compared to conventional exercise programs for rehabilitation. Cyberpsychol Behav.

[B6] Merians AS, Jack D, Boian R, Tremaine M, Burdea GC, Adamovich SV, Recce M, Poizner H (2002). Virtual reality-augmented rehabilitation for patients following stroke. Phys Ther.

[B7] Girone M, Burdea G, Bouzit M, Popescu V, Deutsch JE (2000). Orthopedic rehabilitation using the "Rutgers ankle" interface. Studies in Health Technology & Informatics.

[B8] Rothbaum BO, Hodges LF, Kooper R, Opdyke D, Williford JS, North M (1995). Effectiveness of computer-generated (virtual reality) graded exposure in the treatment of acrophobia. Am J Psychiatry.

[B9] Viirre E (1996). Vestibular telemedicine and rehabilitation. Applications for virtual reality. Studies in Health Technology & Informatics.

[B10] Viirre E, Draper M, Gailey C, Miller D, Furness T (1998). Adaptation of the VOR in patients with low VOR gains. J Vestib Res.

[B11] Kramer PD, Roberts DC, Shelhamer M, Zee DS (1998). A versatile stereoscopic visual display system for vestibular and oculomotor research. J Vestib Res.

[B12] Herdman SJ, Whitney SL, Herdman SJ (1999). Treatment of vestibular hypofunction. Vestibular Rehabilitation.

[B13] Fetter M, Zee DS (1988). Recovery from unilateral labyrinthectomy in rhesus monkey. J Neurophysiol.

[B14] Miles FA, Eighmy BB (1980). Long-term adaptive changes in primate vestibuloocular reflex. I. Behavioral observations. J Neurophysiol.

[B15] Pavlou M, Lingeswaran A, Davies RA, Gresty MA, Bronstein AM (2001). Machine-based vs. customized rehabilitation for the treatment of chronic vestibular disorders. In ISPG Symposium Maastrict, The Netherlands.

[B16] Shepard NT, Telian SA, Smith-Wheelock M (1990). Habituation and balance retraining therapy. A retrospective review. Neurol Clin.

[B17] Jacob RG, Woody SR, Clark DB, Lilienfeld SO (1993). Discomfort with space and motion: A possible marker of vestibular dysfunction assessed by the situational characteristics questionnaire. Journal of Psychopathology & Behavioral Assessment.

[B18] Bronstein AM (1995). The visual vertigo syndrome. Acta Otolaryngol (Stockh).

[B19] Emmelkamp PM, Krijn M, Hulsbosch AM, de Vries S, Schuemie MJ, van der Mast CA (2002). Virtual reality treatment versus exposure in vivo: a comparative evaluation in acrophobia. Behav Res Ther.

[B20] Cruz Neira C, Sandin Daniel J, DeFanti Thomas A (1993). Surround-screen projection-based virtual reality: the design and implementation of the CAVE. Proc-ACM-SIGGRAPH-93-Conf-Comput-Graphics Publ by ACM, New York, NY, USA.

[B21] Hettinger Lawrence J, Riccio Gary E (1992). Visually induced motion sickness in virtual environments. Presence: Teleoperators and Virtual Environments.

[B22] Pausch R, Crea T, Conway M (1992). A literature survey for virtual environments: military flight simulator visual systems and simulator sickness. Presence: Teleoperators and Virtual Environments.

[B23] Jacobson J, Redfern MS, Furman JF, Whitney SL, Sparto PJ, Wilson JB, Hodges LF (2001). Balance NAVE: a virtual reality facility for research and rehabilitation of balance disorders. Proceedings of the ACM symposium on virtual reality software and technology: 2001; Banff, Alberta, Canada.

[B24] Kennedy RS, Lane NE, Berbaum KS, Lilienthal ML (1993). Simulator sickness questionnaire: an enhanced method for quantifying simulator sickness. International Journal of Aviation Psychology.

[B25] Baltzley DR, Kennedy RS, Berbaum KS, Lilienthal MG, Gower DW (1989). The time course of postflight simulator sickness symptoms. Aviat Space Environ Med.

[B26] Cobb SVG, Nichols S, Ramsey A, Wilson JR (1999). Virtual reality-induced symptoms and effects (VRISE). Presence: Teleoperators and Virtual Environments.

[B27] Regan EC, Price KR (1994). The frequency of occurrence and severity of side effects of immersion virtual reality. Aviat Space Environ Med.

[B28] Kennedy RS, Stanney KM, Dunlap WP (2000). Duration and exposure to virtual environments: sickness curves during and across sessions. Presence: Teleoperators and Virtual Environments.

[B29] Stanney KM, Kennedy RS, Drexler JM, Harm DL (1999). Motion sickness and proprioceptive aftereffects following virtual environment exposure. Appl Ergon.

[B30] Mon-Williams M, Wann JP, Rushton S (1993). Binocular vision in a virtual world: visual deficits following the wearing of a head-mounted display. Ophthalmic & Physiological Optics.

[B31] Rushton SK, Riddell PM (1999). Developing visual systems and exposure to virtual reality and stereo displays: some concerns and speculations about the demands on accommodation and vergence. Appl Ergon.

[B32] DiZio P, Lackner JR (1992). Spatial orientation, adaptation, and motion sickness in real and virtual environments. Presence: Teleoperators and Virtual Environments.

[B33] Howarth PA, Finch M (1999). The nauseogenicity of two methods of navigating within a virtual environment. Appl Ergon.

